# Why Do I Feel More Confident? Bandura's Sources Predict Preservice Teachers' Latent Changes in Teacher Self-Efficacy

**DOI:** 10.3389/fpsyg.2016.01486

**Published:** 2016-10-19

**Authors:** Franziska Pfitzner-Eden

**Affiliations:** Department of Education and Psychology, Freie Universität BerlinBerlin, Germany

**Keywords:** teacher self-efficacy, sources, preservice teachers, practicum, latent-true-change

## Abstract

Teacher self-efficacy (TSE) is associated with a multitude of positive outcomes for teachers and students. However, the development of TSE is an under-researched area. Bandura (1997) proposed four sources of self-efficacy: mastery experiences, vicarious experiences, verbal persuasion, and physiological and affective states. This study introduces a first instrument to assess the four sources for TSE in line with Bandura's conception. Gathering evidence of convergent validity, the contribution that each source made to the development of TSE during a practicum at a school was explored for two samples of German preservice teachers. The first sample (*N* = 359) were beginning preservice teachers who completed an observation practicum. The second sample (*N* = 395) were advanced preservice teachers who completed a teaching practicum. The source measure showed good reliability, construct validity, and convergent validity. Latent true change modeling was applied to explore how the sources predicted changes in TSE. Three different models were compared. As expected, results showed that TSE changes in both groups were significantly predicted by mastery experiences, with a stronger relationship in the advanced group. Further, the results indicated that mastery experiences were largely informed by the other three sources to varying degrees depending on the type of practicum. Implications for the practice of teacher education are discussed in light of the results.

## Introduction

Researchers have identified a multitude of meaningful associations between teacher self-efficacy (TSE) and a range of sought after outcomes for inservice and preservice teachers, as well as for students. For inservice teachers, for example, resilience (Beltman et al., 2011), instructional quality (Holzberger et al., 2013), occupational commitment (Klassen and Chiu, 2011; Chesnut and Burley, 2015), job satisfaction (Klassen and Chiu, 2010), teaching performance (Klassen and Tze, 2014), and burnout (Schwarzer and Hallum, 2008) have been documented to be linked to TSE. For preservice teachers, for example, burnout (Fives et al., 2007), occupational commitment (Klassen and Chiu, 2011; Chesnut and Burley, 2015), and commitment to finishing a teaching degree (Pfitzner-Eden, 2016) have been found to be associated with TSE. Moreover, TSE has been shown to be positively related to students' academic achievement (Caprara et al., 2006; Klassen and Tze, 2014).

In contrast, studies focusing on factors that can predict TSE development are rare (e.g., Henson, 2002; Klassen et al., 2011). For years, reviewers (Henson, 2002; Klassen et al., 2011) have highlighted a lack of studies regarding the formation of TSE beliefs as one key problem hampering progress in this field of research. In light of the predictive power of TSE beliefs, systematically studying the formation of these beliefs seems warranted. Furthermore, a systematic study would constitute a necessary first step toward offering guidance to teacher educators, who are interested in fostering the TSE beliefs of preservice teachers. Supporting the development of TSE beliefs during initial teacher preparation is of particular importance because failures are especially detrimental to self-efficacy development if they occur early on Bandura (1997).

According to Bandura (1997), individuals form self-efficacy beliefs by interpreting information regarding their own capabilities. This information stems from four sources: mastery experiences, vicarious experiences, verbal persuasion, and physiological and affective states. Mastery experiences provide information about one's successes, but also failures. Generally, successful experiences increase self-efficacy beliefs, while experiences of failure lower them. Vicarious experiences provide information about modeled attainments of others, which influence one's self-efficacy beliefs by demonstrating and transferring competencies (model learning) and by providing a point of reference for social comparison. Verbal persuasion by “significant others” (Bandura, 1997; p. 101) can convince people of their capabilities, especially if this persuasion comes from a credible source. Physiological and affective states provide information about physiological and affective arousal during situations in which the capability in the domain in question is demonstrated. In stressful situations people tend to read this somatic information as an indicator of dysfunction, thus impacting negatively on self-efficacy beliefs.

Bandura emphasizes the importance of a cognitive processing stage, at which the information from each source is interpreted and integrated, whereby different weights are assigned to the sources. Among the four sources, mastery experiences generally have the strongest effect on self-efficacy development, because they are the most authentic indicators of one's capabilities (e.g., Bandura, 1997). Consequently, previous studies on the development of TSE have focused on the teaching practicum (or field experience) at a school (e.g., Hoy and Woolfolk, 1990; Fives et al., 2007; Knoblauch and Woolfolk Hoy, 2008; Klassen and Durksen, 2014), as it provides an ideal opportunity to gather mastery experiences and thus effect changes in TSE. However, because there have been no reliable measures of the sources for TSE so far (Klassen et al., 2011), a systematic study of the sources and how they contribute to TSE development has not yet been possible. The present study addresses this gap by providing an instrument to assess the four theorized sources of TSE. Confirmatory factor analysis (CFA) was used to test the construct validity of this new measure. In order to examine the predictive validity of this instrument, it was explored whether each of the four sources significantly predicted latent TSE changes that occurred during a practicum. Data were assessed in two groups of preservice teachers at different stages of a teacher preparation program (beginning vs. advanced), which allowed for a comparison of two different types of practicum format (observation vs. teaching), in which the sources might impact differently on TSE development. In order to explore how preservice teachers might integrate the information from the four different sources, three plausible models of interrelationships among the sources were tested.

### Teacher self-efficacy and its development

The theoretical framework underpinning this work is Bandura's conception of the self-efficacy construct, which is a central feature in his social cognitive theory (Bandura, 1977, 1986, 2001). Bandura defines self-efficacy as the “beliefs in one's capabilities to organize and execute the courses of action required to produce given attainments.” (Bandura, 1977, p. 3). Bandura (1997) has put forward manifold evidence of the strong predictive power of self-efficacy beliefs. According to Bandura (1997, 2006), self-efficacy influences behavior via determining what goals and challenges individuals set for themselves, how much effort they choose to invest in pursuing their goals and overcoming challenges, and to what extent they persist in the face of difficulties and obstacles. TSE, specifically, can be understood as the beliefs that inservice and preservice teachers hold about their capabilities to organize and execute the courses of action required to produce given teaching attainments with regard to instruction, classroom management, and student engagement. Tschannen-Moran and Woolfolk Hoy (2001) first operationalized TSE as encompassing these three dimensions. Their three-dimensional conception has been widely adopted by researchers in this field (e.g., Duffin et al., 2012).

TSE beliefs are understood to be most malleable early on in a teacher's career, during teacher education (Woolfolk and Hoy, 1990; Henson, 2002). For this reason, research on the development of TSE has generally focused on preservice teachers, and more specifically focused on the practicum experience. On the one hand, this is due to the practicum being considered a “critical, influential, and transformational stage” (Klassen and Durksen, 2014, p. 168), while on the other hand it is due to the hypothesized importance of mastery experiences. This line of research has produced mixed results with studies reporting increases (Hoy and Woolfolk, 1990; Woolfolk Hoy and Burke Spero, 2005; Fives et al., 2007; Knoblauch and Woolfolk Hoy, 2008; Klassen and Durksen, 2014) and decreases in TSE (Lin and Gorrell, 2001; Pendergast et al., 2011; Garvis et al., 2012). However, it is unclear what weight these results carry, because some of these studies have been methodologically hampered. For example, Garvis et al. (2012), Lin and Gorrell (2001), Hoy and Woolfolk (1990), and Pendergast et al. (2011) compared pre-post group means rather than analyzing within-person change. However, differences in group means are no indication of within-person change (e.g., McArdle, 2009), and thus do not offer any insights into TSE development (for a discussion of methodological drawbacks in research on TSE development see also Pfitzner-Eden, 2016). On the other hand, the inconclusive results might also reflect the sources' potential to cause either positive or negative change.

Bandura (1997) theory of the sources can be applied to explain how the development of TSE in preservice teachers takes place during a practicum at a school. *Mastery experiences* (i.e., successes or failures) which are generated in an actual classroom should have the strongest effect on TSE development, because these experiences provide genuine evidence of whether or not preservice teachers can accomplish the task in question, for example, independently teaching a class or aiding an experienced teacher in organizing group work. The practicum also offers many opportunities for *vicarious experiences*. Observing classes of experienced teachers provides preservice teachers with an opportunity for model learning. This is particularly beneficial for TSE development when several competent teachers can be observed overcoming difficult situations. In addition, vicarious experiences are hypothesized to exert a greater influence on self-efficacy formation when people have little prior experience in the domain to be evaluated. If the practicum experience is supervised by a mentor teacher at the placement school, the mentor would act as a strong source of *verbal persuasion*. The impact the mentor teacher can exert on the TSE development of preservice teachers depends on the perceived credibility of the mentor. This credibility is high, when mentors are themselves competent teachers, are experienced in judging the accomplishments of different preservice teachers, and are knowledgeable with regard to the task-related demands that preservice teachers face. The practicum also offers maybe the first authentic opportunity for preservice teachers to experience a range of somatic indicators or *physiological and affective states*. Such indicators are particularly relevant in informing TSE beliefs, if the domain of functioning includes stressful or taxing situations. Since the practicum is considered to be a very stressful part of teacher preparation (e.g., Klassen and Durksen, 2014), it carries the potential to negatively affect TSE development.

The cognitive processing stage is paramount in interpreting and integrating the information from the sources and thus in informing changes in self-efficacy beliefs (Bandura, 1997). With regard to interpreting source information, for example, physiological and affective states in the form of physical arousal (e.g., sweating, increased heart rate) in a teaching situation can be interpreted as anxiety in the face of the teaching task and attributed to one's own inadequacy, or it can be interpreted as a common reaction to the teaching task that does not necessarily convey any negative information about one's capability. In the latter instance, TSE beliefs would not be affected negatively, whereas in the former instance they would be negatively affected.

Specifically concerning mastery experiences, Bandura (1997) states that “Changes in perceived efficacy result from cognitive processing of the diagnostic information that performances convey about capability rather than from the performances *per se*.” (p. 79). However, if the domain of functioning is complex and people have little prior experience in the domain to be evaluated, then interpreting how successful performances are is not straightforward. Because teaching is a complex task (e.g., Darling-Hammond and Bransford, 2005) and preservice teachers have little prior knowledge of the actual demands and complexities of the teaching task (Tschannen-Moran et al., 1998), preservice teachers judgment of mastery experiences might be informed not only by their performances *per se* but also to some degree by the other sources. For example, according to Bandura (1997), when interpreting whether a performance was successful, people take task difficulty into account. But in order to gauge “the difficulty of tasks, people often fall back on normative information about the success rates of others” (Bandura, 1997, p. 83)—so people use vicarious experiences to inform their judgment of mastery experiences. However, task difficulty and by extension mastery experiences could also be informed by a mentor teacher, which could, for example, let a preservice teacher know that teaching a particular class is very difficult, or that other preservice teachers have also fared badly on a similar task.

Regarding the integration of information from the four sources, Bandura (1997) provides no specific guidelines. He states that “the weights assigned to different types of efficacy information may vary across different domains of functioning” (p. 114), thus in order to examine the formation of TSE, it is warranted to study the sources specifically for the domain of teaching. Bandura (1997) further states that the sources “vary in their informativeness and degree of interrelatedness” (p. 114), that the sources “vary in the complexity of their relations to” (p. 114) self-efficacy judgments, and that the sources “can also be combined configurally” (p. 114), whereby the weight of one source depends on other sources. When translating these statements into data analysis terms, this opens up a range of possibilities of relationships among the sources (i.e., correlation, moderation, mediation).

### Previous research on the sources of teacher self-efficacy

So far, only one quantitative measure of the sources of TSE has been published in a peer-reviewed journal, the Teaching Efficacy Sources Inventory (TESI) by Poulou (2007). The TESI was derived using an inductive qualitative approach, whereby the author analyzed statements by preservice teachers regarding factors that would influence and promote their sense of teaching efficacy. Among other factors (e.g., motivation), the TESI also reflects the four sources, whereby mastery experiences and verbal persuasion, however, were combined into one factor. Among the sources, this combined factor was found to be the only one to predict the level of TSE. Using an adapted version of Poulou's (2007) TESI and focusing on TSE for classroom management, O'Neill and Stephenson (2011) found that only physiological and affective states (negatively) predicted the level of classroom management TSE in preservice teachers. Considering the combined mastery experiences and verbal persuasion factor as well as the unsuccessful prediction of TSE levels, it can be concluded that the TESI cannot be deemed a reliable and valid instrument to assess the four hypothesized sources of TSE in line with Bandura's conception.

In the absence of reliable and valid measures of the sources of TSE, some researchers used proxies for assessing the sources. These proxy indicators generally feature only one item/indicator and often show little overlap with Bandura's conception. Proxies for mastery experiences that researchers have previously used include: participants' ratings of how satisfied they were with their professional performance (Woolfolk Hoy and Burke Spero, 2005; Tschannen-Moran and Woolfolk Hoy, 2007), participants' ratings of own success compared to peers (Woolfolk Hoy and Burke Spero, 2005), and teaching experience in years (Ruble et al., 2011). “Teaching experience in years” is an example for little overlap with Bandura's conception, as it is an objective indicator that lacks an evaluative component and thus does not reflect the interpretation that takes place at the cognitive processing stage. Furthermore, the rating of participants' success compared to peers confounds two sources: mastery and vicarious experiences. The results from these studies confirm the incongruence with Bandura's conception. While neither “teaching experience in years” nor the confounded success rating showed a relationship with TSE, participants' satisfaction with their own performance was significantly related to TSE.

Proxy measures for vicarious experiences were not reported in any studies published in peer-reviewed journals. Proxies for verbal persuasion that researchers have previously used include preservice teachers' ratings of support provided by: their mentor during student teaching (Moulding et al., 2014); school leaders (Ruble et al., 2011); colleagues, school administrators, parents, and the community (Woolfolk Hoy and Burke Spero, 2005; Tschannen-Moran and Woolfolk Hoy, 2007). While perceived support may in some cases also be indicative of verbal persuasion, it is by no means always an indicator of whether preservice teachers received verbal persuasion regarding their capabilities. Consequently, these ratings are not closely aligned with Bandura's (1997) description of this source. Again, the results from these studies support this assumption, as the proxies for verbal persuasion produced inconclusive results, with only two studies (Woolfolk Hoy and Burke Spero, 2005; Moulding et al., 2014) reporting the expected positive relationship to TSE. A proxy indicator for physiological and affective states was only assessed in one study: self-reported levels of burnout (Ruble et al., 2011). Not surprisingly, the authors did find the expected negative association with TSE. However, this is a well-established relationship, whereby there is some convincing evidence that TSE is a predictor of burnout, rather than the other way around (Schwarzer and Hallum, 2008). More importantly, it is questionable to what degree burnout can be considered an indicator of physiological and affective states that is consistent with Bandura's description.

With the exception of one study (Woolfolk Hoy and Burke Spero, 2005), all reviewed quantitative studies on the sources of TSE have either correlated or predicted TSE levels. This is a common way of establishing convergent validity for the sources which researchers (e.g., Usher and Pajares, 2009) in other fields have also applied when testing their source instruments. Nonetheless, predicting TSE *changes* instead of *states* would be an analysis approach that could actually test whether the sources predicted development in TSE. According to Bandura (1997) the sources should predict levels of self-efficacy, but moreover the sources are theorized to cause changes (i.e., development) in self-efficacy beliefs. However, this can only be tested by relating changes in TSE to the sources. So far, TSE changes were only predicted in one study (Woolfolk Hoy and Burke Spero, 2005), albeit using manifest change scores that are associated with serious methodological drawbacks (Cronbach and Furby, 1970). For this reason, the current study uses a latent approach.

None of the previous quantitative studies examined how the information from the sources is integrated. However, Tschannen-Moran et al. (1998) conceptualized physiological and affective states as influencing TSE via mastery experiences, and Woolfolk Hoy and Burke Spero (2005) conceptualized mastery experiences as being based on vicarious experiences, albeit with little empirical success, likely because they assessed both sources in one item. Nevertheless, qualitative research has provided some insights with regard to the integration issue. Klassen and Durksen (2014) analyzed qualitative data on preservice teachers' TSE changes during a teaching practicum. Some of the verbatim examples reported in this study indicate that preservice teachers draw on feedback (i.e., verbal persuasion) by their mentor teacher to inform the judgment of their mastery experiences. For example, a participant in Klassen and Durksen's (2014) study said “I have been doing a lot of marking this week and it's building my confidence” (p. 165)—in this case marking is a mastery experience. Another participant stated that he or she was “asking my mentor teacher for help to know I am marking correctly” (p. 165)—this participant engaged in the same task (i.e., marking) as the former participant. However, in order to judge whether this marking was done correctly, this participant did not rely on his or her own performance appraisal, most likely because this task was attempted for the first time. Instead, the participant used feedback from their mentor (i.e., verbal persuasion) to inform the appraisal of his or her own performance on this task (i.e., mastery experience). In Mulholland and Wallace's (2001) case study of an inservice teacher, mastery experiences and verbal persuasion were likewise closely linked. There was also some indication that verbal persuasion by students was used to inform mastery experiences during preservice teaching experiences (p. 249). Morris and Usher (2011) interviewed research professors who identified mastery experiences and verbal persuasion as the most influential sources, whereby both sources were again thought to be closely related.

In addition, results from qualitative research on the sources underline the importance of verbal persuasion by the mentor teacher as an influential source during the practicum (Klassen and Durksen, 2014). This makes sense, because mentor teachers should be perceived as a very credible source. However, it is unlikely that during the practicum the mentor teacher is the only credible source of verbal persuasion. Mulholland and Wallace's (2001) results show students to be another source of verbal persuasion. Source instruments in the domain of academic self-efficacy of students have typically assessed verbal persuasions provided by peers, parents, and teachers (cf. Usher and Pajares, 2009). Yet so far, there is no systematic information on which other sources of verbal persuasion influence TSE development during the practicum. Regarding the other sources, evidence from case studies with inservice teachers confirm the importance of mastery experiences (e.g., Milner and Woolfolk Hoy, 2003), particularly during preservice teaching practicums (Mulholland and Wallace, 2001). Furthermore, (the lack of) vicarious experiences and physiological and affective states were identified as impacting negatively on TSE development (Mulholland and Wallace, 2001).

Since there is little quantitative research on the sources specifically of TSE, it might be useful to take a look into research on the sources of self-efficacy in another more researched domain. Usher and Pajares (2008) provide a comprehensive review of research on the sources of self-efficacy in school, in which they conclude that mastery experiences consistently predict self-efficacy of students, but that evidence for the other three sources was less consistent. The authors point out that so far the quantitative assessment of the sources can only be regarded as preliminary. Usher and Pajares (2008) highlight several shortcomings which also apply to the field of TSE research. Most notably, aggregate scores for more than one source that mask each source's contribution (as in the TESI by Poulou, 2007) and inconsistencies with Bandura's (1997) theoretical guidelines. One such inconsistency is the lack of an evaluative component particularly in mastery experience items.

Taken together, previous research on the four sources of TSE has (a) applied inadequate quantitative measures, (b) confirmed mastery experiences as influencing TSE beliefs, (c) shown a strong relationship between mastery experiences and verbal persuasion, (d) produced few insights regarding vicarious experiences and physiological and affective states, (e) underscored the significance of practical phases during teacher education, (f) provided no systematic information on who is a source of verbal persuasion during the practicum, (g) focused on predicting levels (i.e., state), rather than changes (i.e., development), of TSE. The current study was designed to address several shortcomings of the previous research in this area.

### The current study

The purpose of the current study is two-fold: (1) to present an instrument for assessing the four sources of TSE in close alignment with Bandura's conception of the sources; and (2) to examine how the information of the four sources is integrated when predicting the development of TSE during a school practicum.

#### Validating the source instrument

To achieve maximum consistency with Bandura's (1997) description of the four sources, a deductive approach was used in the development of this measure. In order to examine the reliability and validity of this instrument, it was administered to two groups of preservice teachers (beginning and advanced) who had completed a practicum at a school. The practicum format for the beginning preservice teachers focused on observing teaching, while the format for the advanced preservice teachers focused on teaching independently. In addition to a range of reliability indicators, construct validity was evaluated using confirmatory factor analysis (CFA). To provide first convergent validity information for the new source measure, relationships between the sources and TSE changes during the practicum were inspected. If close alignment with self-efficacy theory was achieved, all sources should significantly predict TSE changes. Furthermore, mastery experiences, in comparison to the other sources, should show the strongest association with TSE changes, as Bandura's (1997) described this as the most influential source. Vicarious experiences and verbal persuasion should be positively, and physiological and affective states should be negatively related to TSE changes. Another indicator of convergent validity would be if both practicum formats differed with respect to the impact the sources exerted over the development of TSE. Specifically, one could expect vicarious experiences to have a greater impact during the observation practicum in the beginning group, and mastery experiences to have a greater impact during the teaching practicum in the advanced group.

Due to the lack of information about who “significant others” are that also act as sources of verbal persuasion during the practicum, the source measure in the current study should be viewed as incomplete. In order to inform further development of this measure, participants in the current study were asked to indicate who else provided them with feedback during the practicum.

#### Exploring source integration

The second purpose of the current study was to explore the following research question: How do preservice teachers integrate the information from the four sources when developing TSE beliefs? Based on Bandura's (1997) description of the integration process and previous research, three plausible hypotheses are derived and then tested using structural equation modeling (SEM).

- *Hypothesis 1*: All sources are simply interrelated and directly predict TSE changes = the *direct model*. Following this hypothesis, no specific assumptions about the relationships among the sources are tested in this model.
- *Hypothesis 2*: Preservice teachers base their appraisal of mastery experiences on verbal persuasions provided by significant others during their practicum = the *partial mediation model*. In this model, it is hypothesized that mastery experiences, vicarious experiences, and physiological and affective states predict TSE changes directly, while verbal persuasion predicts TSE changes indirectly via mastery experiences. This hypothesis reflects previous qualitative insights regarding the role of verbal persuasion (Mulholland and Wallace, 2001; Klassen and Durksen, 2014).
- *Hypothesis 3*: Preservice teachers base their appraisal of mastery experiences during their practicum on verbal persuasions provided by significant others, on their observation of other teachers in their practicum, and on their physiological and affective responses to their own teaching, or to teaching related tasks in the case of beginning preservice teachers = the *full mediation model*. In this model, only mastery experiences predict TSE changes directly, while vicarious experiences, verbal persuasion, and physiological and affective states predict TSE changes indirectly via mastery experiences. This hypothesis also reflects the above-mentioned qualitative insights regarding the role of verbal persuasion, but is additionally based on theoretical conceptions by Tschannen-Moran et al. (1998) and Woolfolk Hoy and Burke Spero (2005).

## Methods

### Participants and procedure

Participants were two groups of German preservice teachers at two different stages of a teacher education program. This program consists of a 3-year Bachelor degree, which is followed either by a 1-year or 2-year Master degree for elementary/middle school teachers or secondary school teachers, respectively. Participants represented a broad range of academic subjects (e.g., mathematics, French, physics, English, geography, chemistry).

Participants in Sample 1 were beginning preservice teachers at an early stage of their Bachelor degree (19% elementary/middle school). Data for Sample 1 were collected at the end of their first year (T1: *N* = 359) and 3 months later at the beginning of their second year of Bachelor study (T2: *N* = 226). Between T1 and T2, participants in Sample 1 completed a 1-month observation practicum at a school. Participants in Sample 2 were advanced preservice teachers, either enrolled in the Master degree (80%), or in their last year of Bachelor study (20%) (31% elementary/middle school). In order to increase numbers in Sample 2, data were collected from two subsequent seminar cohorts. In the first cohort, data were collected at the end of the winter semester (T1: *n*_1_ = 255) and 3 months later at the beginning of the next semester (T2: *n*_1_ = 143). In the second cohort, data were collected at the end of the following summer semester (T1: *n*_2_ = 140) and 3 months later at the beginning of the next semester (T2: *n*_2_ = 80). Both cohorts were comparable with regard to demographic characteristics and initial TSE level. Thus, both cohorts were combined into one group of advanced preservice teachers (T1: *N* = 395; T2: *N* = 223). Between T1 and T2, participants in Sample 2 undertook a 1-month teaching practicum at a school.

Data for each sample were collected in the same university year so that no participants overlapped between Sample 1 and Sample 2. Measures at T1 were administered in a paper and pencil format in seminars that prepared participants for their practicum at the school. At T2, measures had to be administered via an online survey, since the same preservice teachers did not attend another seminar together after the practicum. Participants took on average about 10 min to complete the measures. In compliance with privacy guidelines of the university, participation was voluntary and data were collected anonymously. In order to guarantee anonymity, data had to be matched using a personal code across both times of measurement. The personal code was an anonymous identifier (e.g., first and last letter of the mother's first name) that respondents completed each time. Self-selection bias in entering the study was minimal, as participation at T1 was declined by only three preservice teachers in Sample 1 and five preservice teachers in Sample 2.

### Practicum format

The main task for preservice teachers in the observation practicum was to observe the classroom and teaching of experienced teachers. However, preservice teachers also engaged in a range of other teaching-related tasks (e.g., assisting experienced teachers during lessons). In this practicum format, preservice teachers had a designated mentor teacher at the school, who provided general and professional guidance. The teaching practicum of the advanced group focused on preservice teachers preparing lessons and teaching independently. Preservice teachers in this format also had a designated mentor teacher at the school, who observed their lessons and gave them feedback afterwards. In addition to the mentor teacher at the school, preservice teachers also had a supervisor at the university, who taught the preparatory seminar. This supervisor also observed a lesson taught by the preservice teachers and then provided feedback afterwards. The advanced preservice teachers did also observe lessons of other experienced teachers, but this task was secondary. Irrespective of the practicum format, generally the very experienced and competent teachers at the school are chosen to be mentors. Depending on the size of the school, there might be more than one preservice teacher completing their practicum at the same time.

### Attrition

Descriptive statistics for the longitudinal samples are shown in Table 1. Retention rates between T1 and T2 were 50% for Sample 1 and 42% for Sample 2. While these rates are low, they are comparable to those reported in similar studies with preservice teachers (47% in Malmberg, 2008; 46% in Klassen and Durksen, 2014), or conducted online (44% in Reuter et al., 2010). In order to test attrition bias, T1 characteristics were compared between those that participated at T1 and T2 and those that dropped out between T1 and T2. All means (TSE variables and age) were compared using *t*-tests, whereas χ^2^-tests were used to examine potential differences in gender. In Sample 1, there were no significant differences (all *p*s > 0.05) for all TSE variables. However, males and older participants were more likely to drop out. In Sample 2, there were no significant differences (all *p*s > 0.05) for all TSE variables, as well as gender and age.

**Table 1:** **Longitudinal sample description**.

**Variable at T1**	**T1**	**T1 and T2**	**T1 not T2**
Beginning group	*n* = 359	*n* = 178	*n* = 181
Female	64.4%	70.1%	59.5%
Mean age (SD)	23.80 (5.05)	22.74 (4.75)	24.70 (5.36)
Mean TSE instruction (SD)	6.74 (1.07)	6.79 (1.08)	6.69 (1.06)
Mean TSE classroom management (SD)	6.12 (1.46)	6.22 (1.44)	6.02 (1.47)
Mean TSE student engagement (SD)	6.80 (1.20)	6.87 (1.14)	6.75 (1.26)
Advanced group	*n* = 395	*n* = 167	*n* = 228
Female	67.2%	71.2%	64.3%
Mean age (SD)	26.13 (4.55)	25.86 (4.60)	26.32 (4.51)
Mean TSE instruction (SD)	6.54 (1.10)	6.63 (1.04)	6.48 (1.14)
Mean TSE classroom management (SD)	6.14 (1.42)	6.26 (1.34)	6.05 (1.47)
Mean TSE student engagement (SD)	6.57 (1.16)	6.65 (1.02)	6.53 (1.25)

TSE, teacher self-efficacy; SD, standard deviation.

### Measures

To assess TSE, participants completed the German version of the Scale for Teacher Self-Efficacy (STSE) at T1 and T2 (see Pfitzner-Eden et al., 2014, for reliability and validity estimates of the scale). The STSE is an adapted version of the TSES (Tschannen-Moran and Woolfolk Hoy, 2001), which provides a stable three-dimensional assessment of TSE for preservice teachers at different stages of teacher preparation. Four items each assess TSE for instructional strategies (e.g., “*How certain are you that you can adjust lessons to the proper level for individual students?*”), classroom management (e.g., “*How certain are you that you can control disruptive behavior in the classroom?*”), and student engagement (e.g., “*How certain are you that you can motivate students who show low interest in schoolwork?*”). Participants rate how confident they are regarding their capabilities in the three dimensions using a 9-point response scale ranging from 1 (*not at all certain can do*) to 9 (*absolutely certain can do*).

The sources of TSE were assessed at T2. All items were self-developed (see Table 2 for wording). Item development followed a deductive process in order to achieve maximum alignment with Bandura's theorized sources. Two experts in self-efficacy theory rated the items for their congruence with Bandura's (1997) description of the sources. The self-developed items were pilot tested with a sample of German advanced preservice teachers (*N* = 282) and a sample of New Zealand advanced preservice teachers (*N* = 131). When crafting the items, care was taken to ensure that items did not merely reflect objective indicators, as that would have compromised the cognitive processing stage.

**Table 2:** **Standardized factor loadings, construct reliabilities (CR), manifest means and standard deviations, and internal consistencies (α) of the source items/scales for each group**.

**Source items/scales**	**Beginning cohort (*N* = 226)**	**Advanced cohort (*N* = 223)**
**STANDARDIZED FACTOR LOADINGS**		
**Mastery experiences (CR = 0.87/0.93)**		
I was very satisfied with my own achievements *(in teaching)* during my practicum.	0.95	0.88
During my practicum, I had many successful experiences *(during my own teaching)*.	0.79	0.89
During my practicum, I noted that I could be a very good teacher.	0.63	0.90
I successfully mastered the requirements of the practicum.	0.75	0.82
**Vicarious experiences (CR = 0.93/0.95)**		
I could observe teachers from whom I learned how to be a good teacher.	0.89	0.95
I observed teachers that managed difficult classroom situations successfully.	0.78	0.81
I observed teachers that conducted very good lessons.	0.92	0.94
I could observe teachers from whom I learned a lot.	0.92	0.92
**Verbal persuasion by the mentor (CR = 0.89/0.85)**		
My mentor at school told me that I would be a good teacher.	0.91	0.75
My mentor at school gave me positive feedback.	0.89	0.96
**Verbal persuasion by others (CR = 0.86/0.80)**		
Other people in my practicum told me that I would be a good teacher.	0.85	0.82
Other people in my practicum gave me positive feedback.	0.88	0.81
**Physiological and affective states (CR = 0.83/0.85)**		
During my practicum, I often felt anxious.	0.69	0.75
During my practicum, I mostly felt uncomfortable.	0.76	0.85
During my practicum, I often felt quite down.	0.90	0.83
**MANIFEST MEANS (STANDARD DEVIATIONS)**		
Mastery experiences (α = 0.85/0.93).	7.23 (1.33)	6.99 (1.28)
Vicarious experiences (α = 0.93/0.95).	7.51 (1.50)	6.60 (1.83)
Verbal persuasion by the mentor (α = 0.89/0.83).	6.14 (3.16)	7.19 (2.15)
Verbal persuasion by others (α = 0.86/0.79).	6.18 (2.89)	6.97 (2.14)

The mastery experiences scale comprises four items, whereby two items were not identical across the two samples. In order to accommodate the different nature of the practicum, these items focused on teaching in the advanced group, but were phrased without reference to teaching in the beginning group. The vicarious experiences scale also comprises four items. These items focus on the model learning aspect of this source. Originally four items were constructed for the verbal persuasion scale, but a low internal consistency among the verbal persuasion items indicated that the verbal persuasion items were better conceptualized as two factors reflecting the source of the persuasion: by the mentor teacher and by others. An open response format was included next to this item where respondents could indicate who these “others” were. The physiological and affective states scale comprises three items, which all refer to negative physiological and affective states. Participants rate their agreement using a 9-point response scale ranging from 1 (*not at all true*) to 9 (*exactly true*).

### Analysis

#### Instrument validation

First, a CFA was conducted to examine the construct validity and reliability of the newly developed source measures. A confirmatory, rather than an exploratory, approach was chosen due to the theory-driven nature of the item development. Thus, it was a priori determined which item was supposed to represent which factor (i.e., source). In this case a confirmatory approach is warranted (cf. Brown, 2006). To examine the reliability of the new source measure, internal consistency (Cronbach's α) and construct reliabilities were also computed.

In order to inspect convergent validity, latent true change (LTC) modeling (Steyer et al., 1997) was applied to determine TSE changes that occurred during the practicum (between T1 and T2). LTC models are structural equation models (SEM) that allow for determining changes in the latent true scores (free of measurement error) of repeatedly measured constructs at the intra-individual level. In order to do so, the LTC model uses a latent (i.e., not measured) change score, defined as the part of a score (i.e., TSE at T2) that is not part of that score at the previous measurement (i.e., TSE at T1) (e.g., McArdle, 2009). Once latent change scores are specified, they can be treated like a regular latent construct in SEM, allowing for the introduction of predictor variables (i.e., the sources) that can explain variance in the latent change scores. Since the TSE measure provides a three-dimensional assessment, a general TSE factor was modeled as a second-order factor, whereby the three TSE dimensions served as first-order factors.

Before modeling LTC, scalar measurement invariance (i.e., equal factor structure, factor loadings, and intercepts) over time needs to be demonstrated for all repeatedly measured constructs (e.g., Steyer et al., 1997). In order to test this prerequisite, invariance tests were conducted for the entire hierarchical CFA model of TSE in each sample in the following order recommended by Brown (2006). First, it was tested whether the factorial structure was equal at both assessment points (configural invariance); second, whether the factor loadings were equal (metric invariance); and third, whether the intercepts were equal (scalar invariance) across both times. In order to evaluate invariance, changes in model fit were tested with the χ^2^-difference test for nested models (e.g., Chan, 1998; Palmer, 2001). If the fit does not decrease significantly at each step, invariance can be assumed.

Participants' responses to the open question regarding other sources of verbal persuasion during their practicum were only analyzed descriptively.

#### Model testing

After establishing invariant LTC models, model fit for the competing models was compared as follows. The direct model was compared to the partial and the full mediation model, in order to judge whether mastery experiences acted as a mediator. Then, the partial and full mediation models were compared, in order to judge whether mastery experiences acted as a mediator for all other sources. Next, it was tested whether including a direct path to TSE was necessary (i.e., improved the fit) in the mediation models, or if the entire influence on TSE changes was mediated by mastery experiences. Model fit was compared using the χ^2^-difference test for nested models. Models were nested, as each of the compared models could be derived by constraining parameters of the less restrictive models they were compared with. If a significant difference was found, the less restrictive model should be favored, if there is no significant difference the more restrictive model should be favored (e.g., Schermelleh-Engel et al., 2003).

A multi index strategy (e.g., Hu and Bentler, 1999) was used to evaluate model fit for the LTC models. As an incremental fit index, the Comparative Fit Index (CFI) was inspected, for which Hu and Bentler (1999) recommend a cut-off value of 0.95. Further, as an absolute fit index the Root Mean Square Error of Approximation (RMSEA) was inspected, for which Hu and Bentler (1999) recommend a cut-off value of 0.06. The χ^2^ test-statistic of exact fit is also reported.

In order to reduce the amount of estimated parameters in the model in relation to the fairly small sample size, item parcels were used as indicators of the measurement models for the three latent TSE factors (for a discussion of pitfalls and merits of item parceling see Little et al., 2002). Taking item-to-construct balance into account, two items were parceled to one indicator so that each of the three latent TSE factors featured two indicators. Measurement errors of the second indicators were allowed to correlate between T1 and T2 (Steyer et al., 2000).

*Mplus* Version 7 (Muthén and Muthén, 1998-2012) was used to compute all models. The Full Information Maximum Likelihood estimation method (FIML; see Wothke, 2000 for a detailed discussion) was used, as recommended by Steyer et al. (2000) for longitudinal data with a large number of missings. The FIML method takes into account all information available from the observed data when estimating model parameters, which results in a less biased estimate than other common practices (e.g., listwise deletion, mean-imputation) of dealing with missing data (Wothke, 2000). The significance level was set at *p* < 0.05 for all analyses. As an indicator of effect size, standardized path coefficients can be regarded as the effect size *r* (Durlak, 2009), for which Cohen (1992) suggested to interpret values of 0.10, 0.30, and 0.50 as small, medium and large effects, respectively.

## Results

### Instrument validation

CFA results for the source measure are presented in Table 2. Model fit was good for both the beginning group: CFI = 0.974; RMSEA = 0.055; $\chi_{\left(80,\text{N}=226\right)}^{2}$ = 134.88, *p* < 0.001; and the advanced group: CFI = 0.972; RMSEA = 0.063; $\chi_{\left(80,\text{N}=223\right)}^{2}$ = 148.98, *p* < 0.001. All factor loadings exceeded the 0.60 mark for accurate representation recommended by Guadagnoli and Velicer (1988). All construct reliabilities of the latent source factors were well above the 0.60 value recommended by Bagozzi and Yi (1988), thus indicating an excellent internal consistency of each latent source construct. Internal consistencies, manifest means, and standard deviations for the source scales are also presented in Table 2. Internal consistencies were reasonably high, indicating a good reliability for such brief scales. Factor intercorrelations between source constructs (see Table 3) did not exceed 0.62. This is an indication that the source factors should indeed be treated as distinct factors (Brown, 2006).

**Table 3:** **Latent intercorrelations between all constructs and bivariate correlations/standardized path coefficients and *R*^2^ between the source variables and TSE_2−1_**.

**Source variable**	**ME**	**VE**	**VPm**	**VPo**	**PAS**	**TSE1**	**TSE_2−1_**	***R*^2^**
**BEGINNING COHORT**								
Mastery Experiences (ME)	–						0.48^*^	0.232^*^
Vicarious Experiences (VE)	0.45^*^	–					0.33^*^	0.108^*^
Verbal Persuasion Mentor (VPm)	0.44^*^	0.32^*^	–				0.32^*^	0.104^*^
Verbal Persuasion Others (VPo)	0.39^*^	0.25^*^	0.62^*^	–			0.30^*^	0.880^*^
Physiological and Affective States (PAS)	−0.50^*^	−0.43^*^	−0.29^*^	−0.23^*^	–		−0.35^*^	0.121^*^
TSE1	0.35^*^	0.25^*^	0.15	0.19^*^	−0.13	–	–	–
TSE_2−1_	0.25^*^	0.17	0.26^*^	0.20^*^	−0.28^*^	−0.31^*^	–	–
**ADVANCED COHORT**								
Mastery Experiences (ME)	–						0.51^*^	0.257^*^
Vicarious Experiences (VE)	0.48^*^	–					0.33^*^	0.107^*^
Verbal Persuasion Mentor (VPm)	0.61^*^	0.56^*^	–				0.40^*^	0.163^*^
Verbal Persuasion Others (VPo)	0.48^*^	0.38^*^	0.55^*^	–			0.37^*^	0.135^*^
Physiological and Affective States (PAS)	−0.47^*^	−0.26^*^	−0.31^*^	−0.15	–		−0.35^*^	0.121^*^
TSE1	0.25^*^	0.19^*^	0.13	0.10	−0.36^*^	–	–	–
TSE_2−1_	0.36^*^	0.19^*^	0.26^*^	0.32^*^	−0.09	−0.57^*^	–	–

*TSE_2-1_ = latent change variable; bivariate correlations/standardized path coefficients are reported in the second to last column. The amount of variance in TSE_2-1_ accounted for by each source (R^2^) is presented in the right-hand column*.

* *p < 0.05*.

#### Measurement invariance over time

The model fit for the configural invariance model was very good for both the beginning group: CFI = 0.984; RMSEA = 0.046; $\chi_{\left(42,\text{N}=407\right)}^{2}=$ 78.0, *p* < 0.05; and the advanced group: CFI = 0.978.; RMSEA = 0.051; $\chi_{\left(42,\text{N}=448\right)}^{2}=$ 91.2, *p* < 0.05. Model fit did not decrease significantly between the configural invariance and the metric invariance model for either group: beginning preservice teachers: Δχ^2^ = Δdf = 5; *p* = 0.13; advanced preservice teachers: Δχ^2^ = 4.1; Δdf = 5; *p* = 0.53. Further constraining the intercepts to be equal over time resulted in a non-significant change in model fit: beginning preservice teachers: Δχ^2^ = 5.1; Δdf = 5; *p* = 0.40; advanced preservice Δχ^2^ = 10.2; Δdf = 5; *p* = 0.07. Accordingly, measurement invariance over time was demonstrated for all repeatedly measured constructs in the latent change analysis. The model fit of the final scalar invariance model was identical to the model fit of the LTC model reported in the next section.

#### Convergent validity

Bivariate correlations between the latent source factors and the latent change in TSE are presented in Table 3. As expected, the bivariate correlations were highest between mastery experiences and TSE changes, and all other sources showed the expected direction of relationship. Furthermore, each source predicted a significant amount of variance in TSE changes. Also as expected, mastery experiences accounted for the largest amount of variance in TSE changes. Unexpectedly, the bivariate correlation between vicarious experiences and TSE changes, and the variance in TSE changes accounted for by vicarious experiences was equivalent in both groups. The intercorrelations between the latent factors of the CFA are also presented in Table 3. Intercorrelations between the sources were mostly of medium to large effect size, with few small effects (e.g., between verbal persuasion by others and physiological and affective states). Controlling for initial levels of TSE, intercorrelations between each source and TSE change dropped as compared to the bivariate correlations, reflecting common variance shared among the sources, and between the sources and initial TSE levels. When all sources and initial TSE levels were taken into account, TSE changes in the beginning group showed a weaker association with mastery experiences than in the advanced group.

#### Verbal persuasion by “others”

The response rate to this open question was 52% in Sample 1 and 59% in Sample 2. Answers of participants who responded are presented in Table 4. Students were most likely to be a source of verbal persuasion in both samples, however, even more so in the advanced group. The next most common source of verbal persuasion in both samples were other teachers, meaning teachers that were not the mentor teacher. The next two most common sources of verbal persuasion named by the beginning group were school principals and other school staff (e.g., teacher aides, social workers), while the next two most common sources of verbal persuasion for the advanced preservice teachers were their peers (i.e., other preservice teachers) and university supervisor. Verbal persuasion from peers played a lesser role in the beginning group as compared to the advanced group. A small proportion of beginning preservice teachers even reported feedback from parents, whereby this source was virtually non-existent in the advanced group.

**Table 4:** **List of “others” that acted as a source of verbal persuasion for preservice teachers during the practicum**.

**Others**	**Beginning group (*n* = 118)**		**Advanced group (*n* = 132)**	
	**Count**	**Percent (%)**	**Count**	**Percent (%)**
Students	76	64.4	94	71.2
Other teachers	39	33.1	38	28.8
School principal	25	21.2	11	8.3
Other school staff	15	12.7	3	2.3
University supervisor	–	–	22	16.7
Peers	6	5.1	23	17.4
Parents	4	3.4	1	0.8

Participants could list multiple “others,” thus percentages do not add up to 100.

### Model examination

#### Predicting latent changes in teacher self-efficacy

Due to the substantial intercorrelations between the sources of TSE (see Table 3), all sources were allowed to correlate with each other in the latent change model. Since latent changes are by definition related to their initial level, this correlation was also allowed. The LTC model showed a good fit in the beginning group: CFI = 0.983; RMSEA = 0.043; $\chi_{\left(52,\text{N}=407\right)}^{2}=$ 91.7, *p* < 0.05; and in the advanced group: CFI = 0.976.; RMSEA = 0.048; $\chi_{\left(52,\text{N}=448\right)}^{2}=$ 105.5, *p* < 0.05. The standardized group means of the latent changes were 0.232 for the beginning group and 0.165 for the advanced group.

In a next step, the sources were introduced to the LTC model as predictors of the TSE change according to the different models outlined in the current study section. In each of the competing models, correlations between the initial level of TSE and each source were allowed, since they were meaningfully related (see Table 3—all effect sizes were at least small). This could be expected, as for instance, a preservice teacher who started the practicum with a relatively high level of TSE, might not experience the same amount of anxiety (physiological and affective states) during teaching as somebody who had started the practicum with a lower TSE belief.

**Figure 1:**
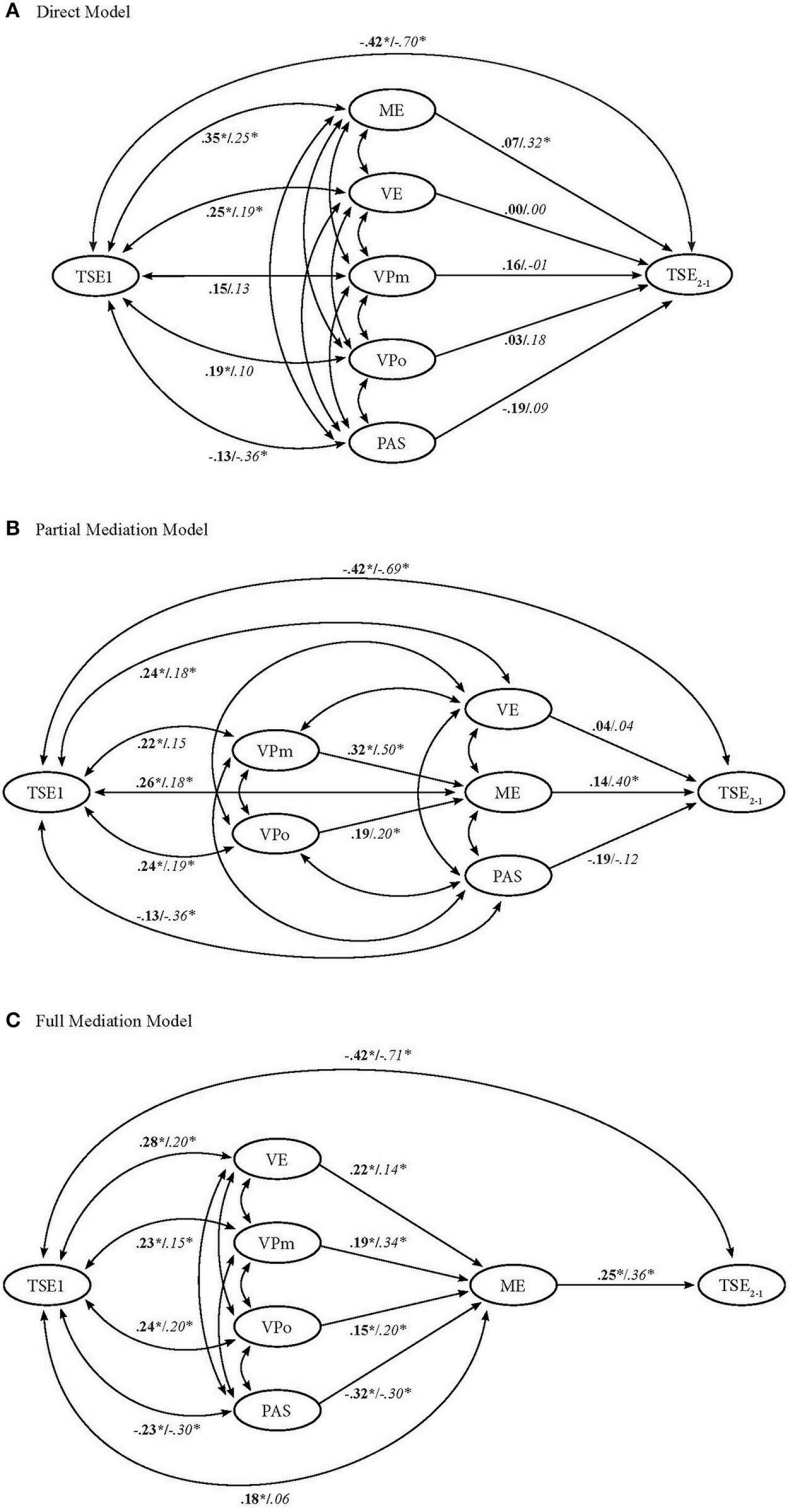
**Structural latent true change model with standardized path coefficients of the competing models for each group of pre-service teachers**. The first coefficients in bold print refer to the beginning preservice teachers, and the second coefficients in italic print refers to the advanced preservice teachers. TSE_2−1_, latent change in teacher self-efficacy; ME, mastery experiences; VE, vicarious experiences; VPm, verbal persuasion by the mentor teacher; VPo, verbal persuasion by “others”; PAS, physiological and affective states. Correlation coefficients between the predictor sources have been omitted from presentation to improve readability. ^*^p < 0.05.

As indicated by all model fit indices in both groups (see Table 5), all three models showed an identical good fit to the data. The results of the χ^2^-difference test for nested models (see also Table 5) showed no significant difference in model fit between the direct model and the more restrictive partial mediation model, indicating that the partial mediation model should be favored over the direct model. There was also no significant difference in model fit between the direct and the more restrictive full mediation model, indicating that the full mediation model should be favored over the direct model. Comparing the mediation models also resulted in no significant difference, indicating that the full mediation model should be favored over the direct model. Further, including paths in each of the mediation models did in no case result in an improved model fit, as indicated by the non-significant χ^2^-difference test for nested models. Since in each case the inclusion of direct paths led to a less restrictive model with equal fit, the more restrictive model (without additional direct paths) should be favored in both cases. To further evaluate the competing models their respective structural models showing the standardized path coefficients are depicted in Figure 1.

**Table 5:** **Model fit for the competing models**.

**Model**	**χ^2^**	***df***	***p***	**CFI**	**RMSEA**	**Δ_χ_**	**Δdf**	**Compared to**
**BEGINNING COHORT**								
Direct model	466.31	302	< 0.001	0.963	0.037	–	–	–
Partial mediation Model	469.29	304	< 0.001	0.963	0.037	2.98 *ns*	2	Direct model
Partial mediation model (with direct effect)^a^	466.31	302	< 0.001	0.963	0.037	2.98 *ns*	2	Partial mediation model
Full mediation model	472.39	306	< 0.001	0.963	0.037	6.09 *ns*	4	Direct model
						3.11 *ns*	2	Partial mediation model
Full mediation model (with directs effects)^a^	466.31	302	< 0.001	0.963	0.037	6.09 *ns*	4	Full mediation model
**ADVANCED COHORT**								
Direct model	491.75	302	< 0.001	0.961	0.037	–	–	–
Partial mediation model	493.53	304	< 0.001	0.961	0.037	1.79 *ns*	2	Direct model
Partial mediation model (with direct effect)^a^	491.75	302	< 0.001	0.961	0.037	1.79 *ns*	2	Partial mediation model
Full mediation model	494.49	306	< 0.001	0.961	0.037	2.74 *ns*	4	Direct model
						0.96 *ns*	2	Partial mediation model
Full mediation model (with directs effects)^a^	491.75	302	< 0.001	0.961	0.037	2.74 *ns*	4	Full mediation model

*CFI, comparative fit index; RMSEA, root mean square error of approximation; ns, non-significant*.

a *All mediation models with direct effects are mathematically identical to the direct model*.

##### The direct model

When all five sources predicted TSE changes simultaneously, all path coefficients were non-significant in the beginning group, and only mastery experiences showed a significant effect on TSE changes in the advanced group. Together, the sources explained 11.7% of the variance in TSE changes in the beginning group, and 16.4% of TSE changes in the advanced group. Since the low weights were somewhat perplexing, but most likely a reflection of the variance shared among the sources, a hierarchical analysis was conducted to further examine this phenomenon. By introducing one source at a time, the unique contribution of each predictor to the variance in TSE change can be determined (e.g., Cohen and Cohen, 1983). The results are reported in Table 6.

**Table 6:** **Hierarchical analysis of the direct effects of the sources on TSE change**.

**Predictor**	**Standardized path coefficient**	***R*^2^**	**Δ*R*^2^**
**BEGINNING PRESERVICE TEACHERS**			
ME	0.24^*^	0.580	–
ME/VE	0.21^*^/0.07	0.630	0.05
ME/VE/VPm	0.15/0.04/0.18	0.860	0.23
ME/VE/VPm/VPo	0.14/0.04/0.16/0.04	0.880	0.02
ME/VE/VPm/VPo/PAS	0.07/0.00/0.16/0.03/−0.19	0.117	0.29
**ADVANCED PRESERVICE TEACHERS**			
ME	0.36^*^	0.128	–
ME/VE	0.34^*^/0.04	0.130	0.02
ME/VE/VPm	0.30^*^/0.01/0.09	0.132	0.02
ME/VE/VPm/VPo	0.28^*^/−0.01/0.00/0.19	0.157	0.25
ME/VE/VPm/VPo/PAS	0.32^*^/0.00/−0.01/0.18/0.09	0.164	0.07

*ME, mastery experiences; VE, vicarious experiences; VPm, verbal persuasion by the mentor; VPo, verbal persuasion by others; PAS, physiological and affective states. Beta weights are reported in the same order as the corresponding predictors. R^2^-values for ME are not identical to those reported in Table 3, as in the analysis here initial levels of TSE are taken into account*.

* *p < 0.05*.

Including mastery experiences as the first predictor is in line with Bandura's (1997) theory of the sources, whereby mastery experiences are assumed to exert the greatest influence over changes in self-efficacy. Indeed, in both groups, mastery experience was the one single predictor that explained the most variance in TSE changes. In the beginning group, the effect of mastery experiences was attenuated with each source added. Particularly adding verbal persuasion by the mentor teacher and physiological and affective states reduced the weight of mastery experiences and improved the prediction of TSE change (Δ*R*^2^). Beta weights are equivalent to partial correlations (e.g., Kline, 2011) and the partial correlation of a predictor × signifies the proportion of the criterion variance that is not associated with the other predictors but is associated with the predictor × (e.g., Cohen and Cohen, 1983). Thus, in the beginning group, while mastery experiences explained a large proportion of the variance in TSE changes, this particular proportion of variance was nearly entirely shared with all other sources (i.e., the beta weight drops to 0.07). In the advanced group, the effect of mastery experiences was much less attenuated by the inclusion of any other source. In this group, particularly verbal persuasion by others made a unique contribution to the prediction of TSE changes.

##### The partial mediation model

In this model the impact of the verbal persuasion sources on TSE changes was modeled to be fully mediated by mastery experiences. Subsequently this model explained less variance in TSE changes, specifically 9.7% in the beginning group, and 14.3% in the advanced group. Changes in TSE were significantly predicted by preservice teachers' rating of their mastery experiences during the practicum in the advanced group only, although there was still a small effect in the beginning group. In sum, both verbal persuasion factors explained 21.4 and 40.6% of variance in the mastery experiences source in the beginning and the advanced group, respectively. Verbal persuasion by the mentor teacher significantly predicted mastery experiences in each sample and did so to a greater degree in the advanced group. Verbal persuasion by others predicted mastery experiences in both groups to virtually the same degree, but the significance level was just missed in the beginning group. Similarly, to the direct model, vicarious experiences did not exert a direct effect on TSE changes in either group. Physiological and affective states exerted a small but insignificant direct effect on TSE changes in both groups of preservice teachers.

##### The full mediation model

In this model the impact of all other sources on TSE changes was modeled to be fully mediated by mastery experiences. Consequently this model explained less variance in TSE changes, specifically 6.2% in the beginning group, and 13.1% in the advanced group. In both samples, changes in TSE were significantly predicted by preservice teachers' rating of their mastery experiences during the practicum. As expected, this effect was smaller in the beginning group (small to medium effect size) than in the advanced group (medium effect size). In sum, the vicarious experiences, verbal persuasion, and physiological and affective states sources predicted 39.8% and 50.5% of variance in the mastery experiences source in the beginning and the advanced group, respectively. Each source significantly predicted mastery experiences in each sample. However, the weights associated with each source predictor were not identical for the two samples. Specifically, vicarious experiences exerted a greater effect on mastery experience in the beginning than in the advanced group. This is in line with the expectation that vicarious experiences should have a greater impact during the observation practicum. Verbal persuasion by the mentor teacher had a smaller effect on mastery experiences in the beginning group, and the largest (medium-sized) effect of all predictors on mastery experiences in the advanced group. Verbal persuasion by others also carried a slightly greater weight in the advanced group. Physiological and affective states exerted a strong (medium-sized) effect on mastery experiences in both groups of preservice teachers.

## Discussion

### Results and implications

The current study presented a new measure of Bandura's (1997) proposed four sources of self-efficacy for TSE. Taken together, the results provide first evidence of good construct validity, convergent validity, and reliability of the self-developed source scales. Surpassing previous research on the sources of TSE, it could be demonstrated that each of the sources predicted latent changes in preservice teachers' TSE. These predictions were generally consistent with Bandura's (1997) conception of the sources, whereby mastery experiences played the largest role in TSE development. A valid and reliable instrument for assessing the sources of TSE has been deemed long needed in the field of TSE research (e.g., Klassen et al., 2011) and should prove fruitful for conducting further studies on TSE development in preservice teachers.

Furthermore, the present study offers a first systematic insight into who, in addition to the mentor teacher, influences preservice teachers' TSE beliefs via verbal persuasion. This provides a solid basis from which to further develop the source measure for verbal persuasion. The results showed that especially students seem to play a noteworthy role. It would then make sense to take this fact into account, for example, when assigning a particular class to preservice teachers during their practicum. Or it might even be useful to brief students in advance, regarding the impact that their feedback could have on preservice teachers.

In addition, it was explored how preservice teachers integrate the information from the four sources when developing TSE beliefs during a practicum. Three competing hypotheses were tested by comparing SEM models. Each model showed the same good fit to the data. The model comparisons provided first evidence in support of the full mediation model. This model showed an equivalent fit to both the less constrained direct model and the partial mediation model, and should thus be favored (e.g., Schermelleh-Engel et al., 2003). Furthermore, including direct paths between the sources predicting mastery experiences and TSE change did not improve model fit. Due to the shared variance among the sources, the direct model, in which all four sources directly predicted TSE changes, showed that the unique contributions of nearly all sources to TSE changes were small. Only mastery experiences in the advanced group made a substantial unique contribution. Similarly, again with the exception of mastery experiences in the advanced group, the partial mediation model also showed that the unique direct contributions of the sources were small.

Taken together, the results from the model comparison provide first evidence in favor of the third hypothesis that preservice teachers' changes in TSE are directly influenced by the mastery experiences they gather during the practicum, while their mastery experiences, in turn, are largely informed by the other three sources. As expected, the contribution of each source differed for the two groups (i.e., practicum formats). Whereas physiological and affective states, vicarious experiences, and verbal persuasion by the mentor teacher had the largest influence on beginning preservice teachers' ratings of mastery experiences, verbal persuasion by the mentor teacher, physiological and affective states, and verbal persuasion by others had the largest effect on advanced preservice teachers' ratings of mastery experiences. In total, the three sources explained 40% of the variation in the mastery experience ratings in the beginning group and about half of the variation in the advanced group. With respect to Bandura's (1997) theory of the sources, this does not mean that there is no influence of the verbal persuasion, vicarious experiences, and physiological and affective states sources on changes in TSE, but rather that their effects on the TSE development of preservice teachers could be entirely mediated by mastery experiences.

Furthermore, the full mediation model showed stronger associations between mastery experiences and TSE changes in the advanced group than in the beginning group, while associations between vicarious experiences and mastery experiences were stronger in the beginning group as compared to the advanced group. Both results are in line with Bandura's (1997) description of the sources, adding more evidence in favor of the validity of the source measures. According to Bandura's description, the mastery experiences in the advanced group should carry a greater weight with regard to TSE development because they are based on actual teaching experiences, which provide more authentic evidence of one's teaching capabilities when compared to the beginning group. Vicarious experiences, on the other hand, are expected to play a greater role in the beginning group, in which preservice teachers had more opportunity to observe other teachers.

Perhaps most interesting to practitioners and teacher educators, this study showed that positive feedback from the mentor teacher has a significant positive influence on the development of preservice teachers' TSE, possibly via the perception of mastery experiences. Again, this result is consistent with Bandura's (1997) description of verbal persuasion as being particularly influential when it comes from somebody who has expert knowledge in the evaluated field: the mentor teacher. Most likely the influence was stronger during the teaching practicum than the observation practicum because mentors could here base their feedback on preservice teachers' actual teaching performance, which would make the feedback more credible. According to Bandura (1997), credibility is another important aspect that increases the effect that verbal persuasion has on self-efficacy. This finding underscores the significant role mentor teachers at schools play in the development of preservice teachers' TSE, particularly during teaching experiences. It further adds to the qualitative evidence put forward by Klassen and Durksen (2014) regarding the significance of verbal persuasion by the mentor teacher on preservice teachers' TSE changes during a teaching practicum. Consequently, one recommendation to teacher educators, interested in supporting preservice teachers' TSE development, would be to prepare mentor teachers at schools with regard to the process of giving feedback to preservice teachers. This preparation can be guided by social cognitive theory (Bandura, 1997), which provides a host of insights into ways to enhance self-efficacy beliefs using verbal persuasion. For example, in order to foster TSE beliefs mentor teachers should phrase performance feedback in terms of achieved progress toward a certain standard. In comparison, feedback phrased with regard to shortfalls from a certain standard is likely to have detrimental effects on TSE development.

Especially interesting in light of the few previous empirical findings regarding physiological and affective states, the current study shows that negative physiological and affective states contributed strongly across both groups to reduced mastery experiences and consequently to a decrease in TSE. Moulding et al. (2014) state that traditional teacher preparation programs generally involve all sources of TSE except physiological and affective states. The current results provide a convincing reason for addressing this gap in teacher preparation. Since Bandura (1997) highlights that physiological and affective states (and the other sources) do not influence self-efficacy beliefs directly but via cognitive processing, it seems worthwhile to formally integrate, for instance, practicing emotional and physiological self-regulation strategies into the practicum experience. This could possibly be implemented as part of reflective processes guided by the mentor teacher in pre- and post-observation conferences before and after first teaching attempts (for a review on mentoring of beginning teachers see Hobson et al., 2009).

Since there are no prior studies quantifying the contributions of each source to TSE changes, it is difficult to judge the practical relevance of the predictive magnitudes seen in the current study. Nonetheless, the large amount of unexplained variance, some of which is due to initial TSE levels, clearly indicates that there are other factors at play in TSE development. As of yet, some researchers have explored the impact of other factors, usually under the umbrella term of context factors (Tschannen-Moran and Woolfolk Hoy, 2007; Knoblauch and Woolfolk Hoy, 2008; Moulding et al., 2014). However, this research has not related those factors to actual *changes* in TSE, but rather to states, which offer no indication of actual TSE development. Consequently, a comparison of the influence exerted by the sources vs. other factors is not yet possible. Nevertheless, in terms of theory development, the current magnitudes provide a benchmark. This benchmark will allow for a comparison of results produced by other factors, as well as the contribution of the four sources produced by other teacher education programs or differently designed practicum experiences.

### Limitations and future directions

The design used in this study is correlational and thus no causality can be inferred between the sources and changes in TSE. A strictly uni-directional causal relationship between the sources and TSE development is unrealistic as predicted by theory (e.g., Tschannen-Moran et al., 1998), and as the correlations between the sources and the level of TSE at T1 in this study demonstrate. However, the timing of the study (i.e., examining TSE development in preservice teachers) might have allowed for minimal effects of TSE on the sources, and maximal effects for the sources on TSE, since TSE is hypothesized to be most malleable by the sources early on (e.g., Woolfolk Hoy and Burke Spero, 2005). While one model of how the sources interact in predicting TSE changes emerged as the most parsimonious model with an identical fit to the other models (and should thus be favored), there could be numerous other relationships between the sources that might explain the data equally well. Future research employing experimental designs would be needed to demonstrate the assumed causal nature among the sources. Since teacher education programs differ considerably across the world (OECD, 2005), examining to what degree results are similar would contribute to validating the present findings or, in any case, to enhancing our understanding of how the sources interact.

Furthermore, there was a small attrition bias with regard to gender and age in the beginning group. This might impact the generalizability of the results from the observation practicum to male and older preservice teachers. While there was no attrition bias with regard to the dependent variables (TSE), it is still possible that changes in TSE that occurred during the practicum were related to retention.

No data from the mentor teachers at the schools were assessed and neither were preservice teachers' ratings of the credibility of their mentor teachers. Assessing the teaching experience of mentor teachers, for example, would have allowed for an objective indicator of the type of models that were observed by preservice teachers. Although, participants did also observe other teachers at their practicum school. Additionally, credibility ratings could have been used as a control, when determining the influence of the verbal persuasion source on TSE changes. Both aspects could have enriched the findings regarding verbal persuasion and vicarious experiences.

The present assessment of the sources is not independent of context and is thus limited in its general application. The developed source scales are designed specifically to be used for preservice teachers after a practicum experience at a school. There are potentially many other aspects of teacher education (e.g., university course, induction year) for which this assessment of the sources would be unsuitable. However, the context-specific design might have made this measure successful. At the same time, this context-specificity also carries another limitation. The wording in two of the four mastery experiences items is not identical in the two samples, thus limiting the comparability of the mastery experiences source between the two groups.

In order to match the same level of generality of the source measure, TSE changes were examined for the higher-order TSE factor, rather than for the three TSE factors (i.e., instructional strategies, classroom management, student engagement). In fact, the analysis was also conducted using the three dimensions of TSE as dependent variables. This showed that the effects of the sources were equivalent across all three dimensions. Nevertheless, researchers might be interested in detecting more dimension-specific effects, which would require the development of dimension-specific source items. For example, mastery experience items could specifically refer to successes in using a certain method of instruction, managing difficult students, or engaging under-performing students. Exploring this avenue could be useful, for example, when evaluating or developing a teacher education program that has a certain profile focusing on one dimension, for example classroom management, which is often reported to be challenging for preservice teachers (e.g., Veenman, 1984). Dimension-specific source items could then be used in determining if this emphasis is reflected in preservice teachers' development of TSE in classroom management over the course of the program.

Building on the findings this study produced regarding who was also a source of verbal persuasion, future studies could expand the verbal persuasion items, for example, to students, other teachers, peers, and the university supervisor. It could then be more systematically determined what influence the feedback of the different “persuaders” has on TSE development. Are there interactions, for example, between feedback given by the mentor teacher at the school and feedback given by the university supervisor? Whose feedback has a stronger influence: that of the students or the university supervisor?

Future research could also focus on how the contribution of each source, or the integration of the information from each source, changes with career stage. A central assumption in this study is that due to a lack of previous mastery experiences, preservice teachers rely on the other three sources to inform their judgment of mastery experiences. This should not hold true for more experienced inservice teachers, who have accumulated an abundance of mastery experiences. Consequently, the direct model may well be valid for inservice teachers. Regarding the contribution of each source, physiological and affective states, for instance, might have a great impact early on in TSE development, but its influence might fade later on when teachers get used to being in front of a class. Determining what weight is associated with each source at different career stages could inform the design of professional development programs aimed at increasing TSE for inservice teachers, or teacher education programs for teacher candidates.

## Ethics statement

The study (Ph. D. proposal and expose) was approved by the first supervisor and then by the PhD committee of the Department of Education and Psychology at the Freie Universität Berlin.

## Author contributions

The author confirms being the sole contributor of this work and approved it for publication.

## Funding

The author gratefully acknowledges support by the German Research Foundation and the OpenAccess Publication Funds of the Freie Universität Berlin.

### Conflict of interest statement

The author declares that the research was conducted in the absence of any commercial or financial relationships that could be construed as a potential conflict of interest.
